# A clinicopathological study of IgG4-related autoimmune hepatitis and IgG4-hepatopathy

**DOI:** 10.1007/s00535-025-02221-3

**Published:** 2025-02-08

**Authors:** Atsushi Tanaka, Kenji Notohara, Maki Tobari, Masanori Abe, Takeji Umemura, Atsushi Takahashi, Akemi Tsutsui, Takanori Ito, Kohichi Tsuneyama, Atsushi Masamune, Ken-ichi Harada, Hiromasa Ohira, Mitsuhiro Kawano

**Affiliations:** 1 Department of Medicine, Teikyo University School of Medicine, 2-11-1, Kaga, Itabashi-Ku, Tokyo, 173-8605 Japan; 2 Department of Anatomic Pathology, Kurashiki Central Hospital, Okayama, Japan; 3 Department of Gastroenterology, Graduate School of Medicine, Juntendo University, Tokyo, Japan; 4 Department of Gastroenterology and Metabology, Ehime University Graduate School of Medicine, Ehime, Japan; 5 Division of Hepatology and Gastroenterology, Department of Medicine, Shinshu University School of Medicine, Nagano, Japan; 6 Department of Gastroenterology, Fukushima Medical University School of Medicine, Fukushima, Japan; 7 Department of Hepatology, Kagawa Prefectural Central Hospital, Kagawa, Japan; 8 Department of Gastroenterology and Hepatology, Nagoya University Graduate School of Medicine, Nagoya, Japan; 9 Department of Pathology and Laboratory Medicine, Tokushima University Graduate School of Biomedical Sciences, Tokushima, Japan; 10 Division of Gastroenterology, Tohoku University Graduate School of Medicine, Sendai, Japan; 11 Department of Human Pathology, Kanazawa University Graduate School of Medicine, Kanazawa, Japan; 12 Department of Hematology and Immunology, Kanazawa Medical University, Kahoku-Gun, Japan

**Keywords:** AIH, IgG4-sclerosing cholangitis, IgG4-related disease, The comprehensive diagnostic criteria

## Abstract

**Background and aim:**

Although IgG4-related autoimmune hepatitis (IgG4-AIH) and IgG4-hepatopathy have been proposed as hepatic phenotypes of IgG4-related disease (IgG4-RD), their definitions and concepts remain insufficiently established. This study aims to conduct a clinicopathological investigation of cases reported as potential IgG4-AIH or IgG4-hepatopathy.

**Methods:**

In previous nationwide epidemiological studies conducted in 2015 and 2018, we registered 1096 cases of IgG4-sclerosing cholangitis (IgG4-SC). Among these, 19 cases were identified as potential IgG4-AIH by the attending physicians, and other 20 cases as potential IgG4-hepatopathy with available liver histology were further evaluated using immunohistochemistry to assess the possibility of IgG4-AIH or IgG4-hepatopathy. For this purpose, we provisionally established diagnostic criteria for IgG4-AIH and IgG4-hepatopathy, primarily based on the comprehensive diagnostic criteria for IgG4-RD, which include IgG4 + cell count > 10/HPF and an IgG4 + /IgG ratio > 40%.

**Results:**

Of the 19 cases, 2 were diagnosed as IgG4-AIH, with IgG4 + cell counts/HPF of 25.3 and 18.7, and IgG4 + /IgG ratios of 310.2% and 53.4%, respectively. Neither storiform fibrosis nor obliterative phlebitis was observed in the liver of these cases, and both responded excellently to corticosteroid treatment. In addition, from other 20 cases, we diagnosed 8 cases as IgG4-hepatopathy, with IgG4-SC and autoimmune pancreatitis being present in 7 and 2 cases, respectively.

**Conclusion:**

This study identified two cases of IgG4-AIH and eight cases of IgG4-hepatopathy. Further studies are necessary to explore the occurrence of IgG4-AIH using these diagnostic criteria in the AIH cohort. The presence of IgG4-hepatopathy may facilitate the diagnosis of IgG4-SC.

## Introduction

IgG4-related disease (IgG4-RD) is a chronic, multisystemic inflammatory disorder that can affect multiple organs simultaneously or heterogeneously [1]. IgG4-RD is characterized by elevated serum IgG4 levels and the presence of enlarged, nodular, or thickened lesions in affected organs, and significant infiltration of IgG4-positive plasma cells along with fibrosis. The revised comprehensive diagnostic criteria for IgG4-RD, updated in 2020, include (1) marked lymphoplasmacytic infiltration and fibrosis, (2) an IgG4/IgG-positive cell ratio of 40% or higher with at least 10 IgG4-positive plasma cells per high-power field (HPF), and (3) characteristic fibrosis, particularly storiform fibrosis or obliterative phlebitis [2]. Diagnosis of IgG4-RD is confirmed when two or more of these three histological criteria are met. In 2019, classification criteria for IgG4-RD were published by the American College of Rheumatology and European League against Rheumatism [3]. These criteria involve a scoring system based on histopathological findings, immunohistochemical staining results, IgG4 concentration, and imaging studies.

With the recognition of IgG4-RD as a distinct clinical entity, cases have been reported that fulfill the diagnostic criteria for classic autoimmune hepatitis (AIH) while exhibiting features characteristic of IgG4-RD, such as elevated serum IgG4 levels and significant infiltration of IgG4-positive plasma cells in the liver. In 2007, Umemura et al. described a case of a 54-year-old woman with classic AIH, where the pre-treatment score, based on the revised diagnostic criteria proposed by the International AIH Group (IAIHG) [4], was 18. Her serum IgG4 level was markedly elevated to 557 mg/mL, and pronounced infiltration of IgG4-positive plasma cells was observed in the liver. The authors introduced the term IgG4-associated AIH to describe this condition, which exhibits features of both classic AIH and IgG4-RD [5]. In 2010, Umemura et al. reported a similar case of a 42-year-old man with compatible clinical manifestations [6]. Subsequent reports of similar cases emerged not only in Japan but also in Turkey, Thailand, and South Korea [7–11]. These five cases shared the common features of 1) they met the revised diagnostic criteria for classic AIH, 2) exhibited elevated serum IgG4 levels, 3) showed marked infiltration of IgG4-positive plasma cells in the liver, and 4) responded well to corticosteroid therapy. Notably, two of the five cases subsequently developed autoimmune pancreatitis (AIP), another form of IgG4-RD in the pancreatitis, further suggesting a strong likelihood of underlying IgG4-RD in these cases. However, none of the patients exhibited an IgG4/IgG-positive cell ratio of 40% or higher, nor did they demonstrate storiform fibrosis or obliterative phlebitis, as outlined in the comprehensive diagnostic criteria for IgG4-RD.

On the other hand, IgG4-hepatopathy is considered a hepatic complication of IgG4-RD, occurring in association with other IgG4-RD, such as AIP and IgG4-related sclerosing cholangitis (IgG4-SC). In 2007, Umemura et al. conducted a comparable study of liver tissue samples from 17 patients with AIP and 63 patients with other liver diseases. The study revealed that the livers of AIP patients exhibited significant damage, characterized by inflammatory cell infiltration in the portal areas, bile duct injury, and abundant infiltration of IgG4-positive plasma cells [12]. The median bilirubin level in these 17 patients was 1.1 mg/dL, indicating that the observed hepatic pathology was not attributable to obstructive jaundice. Importantly, IgG4-hepatopathy is distinct from IgG4-AIH, as it does not meet the diagnostic criteria for classic AIH.

Thus, the new disease concepts of IgG4-related AIH (IgG4-AIH) and IgG4-hepathpathy were proposed following a series of case reports. However, the definition of these conditions has varied across studies, and the prevalence and pathogenesis of the diseases remain unclear, as we previously summarized in our review [13]. To address these uncertainties, we aimed to clarify the clinical and pathological characteristics of IgG4-AIH and IgG4-hepatopathy in Japan by analyzing cases registered in a nationwide survey of IgG4-SC conducted in 2015 and 2018 [14, 15].

## Patients and methods

### Patients

We conducted a nationwide epidemiological survey in 2015 and 2018 to elucidate the current status of IgG4-SC in Japan [14, 16]. In this survey, diagnosis of IgG4-SC was made in each healthcare facility according to the diagnostic criteria proposed in Japan in 2012 [17]*.* In brief, a diagnosis of IgG4-SC is made based on a combination of four clinical items including (a) biliary tract imaging, (b) elevated serum IgG4 level, (c) coexistence of IgG4-RD in other organs, and (d) histological findings compatible with IgG4-RD. Patients with definite or probable diagnoses were registered in the survey, while those with possible diagnoses were excluded [14]. A total of 1096 cases of IgG4-SC were registered, among which 65 cases were diagnosed as IgG4-AIH by the attending physicians. We requested histopathological tissue samples from the relevant facilities, and 19 liver tissue samples were provided. Of these, IgG4 immunostaining was evaluable in 18 cases. In addition, one case suspected of IgG4-AIH, encountered by one of the authors after the nationwide survey, was also included in the current analysis.

**Fig. 1:**
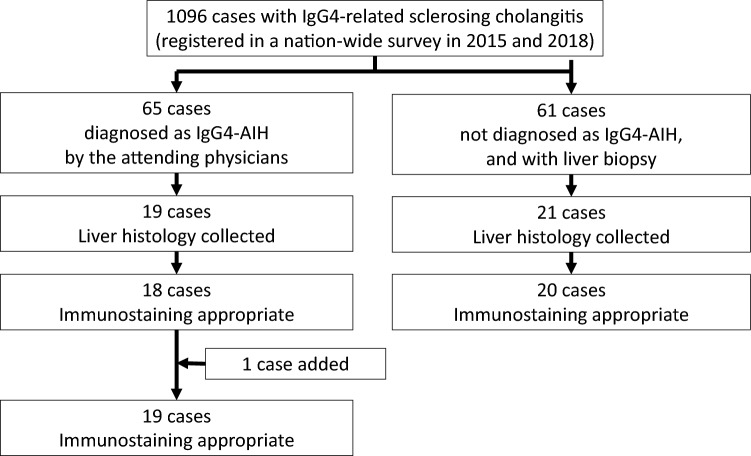
Flowchart of the study. *AIH* autoimmune hepatitis

On the other hand, of the 1096 cases of IgG4-SC, there were 61 cases in which the attending physician did not diagnose IgG4-AIH and a liver biopsy had been performed. We hypothesized that some of these cases might represent IgG4-hepatopathy, and requested the attending physicians to provide liver tissue samples for further analysis. As a result, 21 cases were provided, with IgG4 immunostaining being evaluable in 20 of them. In total, 39 cases were thoroughly examined by a panel of clinical and pathological liver specialists to determine whether they represented IgG4-AIH or IgG4-hepatopathy (Fig. 1). This study was approved by the Teikyo University Medical Research Ethics Committee (18–237) as well as by the Ethics Committee of each participating center.

### Tentative diagnostic criteria of IgG4-AIH and IgG4-hepatopathy

Currently, there are no established diagnostic criteria for IgG4-AIH. The comprehensive diagnostic criteria for IgG4-RD stipulate that the "clinical and imaging" criterion, which requires the presence of characteristic diffuse or localized swelling, masses, nodules, or thickened lesions, must be met for any diagnosis, e.g., definite, probable, or suspected. However, to date, no reported cases of IgG4-AIH have exhibited localized swelling, masses, nodules, or thickened lesions in the liver, suggesting that the comprehensive diagnostic criteria may not be applicable. Furthermore, for a diagnosis of IgG4-AIH, it is crucial that the diagnostic criteria for AIH are also simultaneously fulfilled. Therefore, we have tentatively defined the following criteria for the diagnosis of IgG4-AIH in the current study, as follows:

1. Definite or probable diagnosis of AIH based on the IAIHG Diagnostic Criteria [18]
2. Elevated serum IgG4 (≥ 135 mg/dL)
3. At least two of the following three features in the liver tissues
  - Marked lymphoplasmacytic infiltration with fibrosis
  - IgG4-positive cells > 10/HPF and IgG4/IgG-positive ratio > 40%
  - Typical tissue fibrosis, particularly storiform fibrosis, and/or obliterative phlebitis

In addition, since it is also possible that liver lesions may exist independently, the presence of IgG4-RD in other organs is not considered essential.

Regarding IgG4-hepatopathy, we defined tentative diagnostic criteria as follows:

1. Not meeting the diagnostic criteria of IgG4-AIH
2. Diffuse or focal infiltration of lymphocytes and plasma cells in the portal area
3. IgG4-positive cells > 10/HPF and IgG4/IgG-positive ratio > 40%.

The evaluation of IgG4 and IgG immunostaining was performed according to the consensus statement on the pathology of IgG4-RD [19]. The number of IgG4-positive cells was counted in three fields of view with a high number of IgG4-positive cells, and the average of the IgG4-positive cell counts and the ratio of IgG4-positive cells to IgG-positive cells were calculated.

## Results

### IgG4-AIH

A comprehensive clinical and histopathological analysis of 19 cases of IgG4-AIH, as reported by the attending physicians, revealed that 9 cases were diagnosed as either definite or probable AIH according to the simplified diagnostic criteria. Among the remaining ten cases, two were classified as IgG4-hepatopathy, five as canalicular cholestasis, and three were diagnosed with other diseases; one primary sclerosing cholangitis, and two chronic hepatitis C. In 2 of the 19 cases (AIH-56, AIH-60), IgG4-RD was observed in other organs, suggesting the possibility of IgG4-hepatopathy in the liver tissue.

The histopathological findings of IgG4-AIH in nine cases diagnosed with AIH (AIH-07 to AIH-73) are summarized in Table 1. In three of the nine cases (AIH-07, 12, and 40), IgG4 or IgG immunostaining was either absent or difficult to evaluate, precluding assessment of the IgG4 + /IgG + ratio. In the remaining six cases, three (AIH-14, 29, and 73) met the diagnostic criteria of an IgG4/IgG-positive cell ratio of 40% or greater and 10/HPF or more of IgG4-positive plasma cells. Among these three cases, one case (AIH-29) exhibited a low number of IgG4-positive cells, with an average of 10.1/HPF across three fields of view. These cells were observed only in limited regions of the liver tissue, suggesting a low likelihood of IgG4-AIH in AIH-29. Based on the findings above, only two cases, AIH-14 and AIH-73, met the diagnostic criteria for IgG4-AIH as established in this study.

**Table 1:** Histopathological assessment of potential IgG4-AIH cases

Case #	AIH score*	IgG4 + /HPF	IgG4 + /IgG + ratio (%)	Storiform fibrosis	Obliterative phlebitis	OOI of IgG4-RD
AIH-07	6	16.0	NA	–	–	IgG4-SC
AIH-08	6	1.2	4.2	–	–	IgG4-SC
AIH-12	7	20.2	NA	–	–	IgG4-SC
AIH-14	7	25.3	310.2	–	–	IgG4-SC
AIH-29	6	10.1	71.7	–	–	IgG4-SC
AIH-39	8	4.2	45.5	–	–	IgG4-SC, RPF
AIH-40	9	24.7	NA	–	–	IgG4-SC
AIH-72	7	20.7	19.0	–	–	IgG4-SC
AIH-73	8	18.7	53.4	–	–	IgG4-SC, IgG4-SA

*AIH* autoimmune hepatitis, *AIP* autoimmune hepatitis, *HPF* high-power field, *OOI* other organ involvement, *IgG4-SC* IgG4-related sclerosing cholangitis, *IgG4-RD* IgG4-related disease, *IgG4-SA* IgG4-related sialadenitis, *NA* not assessable, *RPF* retroperitoneal fibrosis

^*^AIH score was calculated using simplified scoring system proposed by the International AIH Group^1^. Probable AIH ≥ 6, definite AIH ≥ 7

AIH-14 is a previously reported case elsewhere [20]. The patient, a female in her 40 s, presented with elevated aminotransferases levels, along with elevated IgG4 (669 mg/dL), and a positive anti-nuclear antibody titer of 1:160. Histopathological analysis revealed prominent lymphoplasmacytic infiltration accompanied by fibrosis. A notable presence of IgG4-positive plasma cells was observed, with 25.3 cells per high-power field and an IgG4/IgG ratio of 310.2%. Although storiform fibrous and obliterative phlebitis were absent, the patient fulfilled the provisional diagnostic criteria for IgG4-AIH defined in the current study. The histological features were consistent with the acute hepatitis phase of AIH, characterized by diffuse distribution of IgG4-positive cells in the portal areas, lobules, and central lobular regions, with a particularly high concentration in the portal areas. The patient was treated with prednisolone (PSL), leading to a rapid and marked reduction in ALT, IgG, and IgG4 levels. She is currently maintained on 5 mg/day, with no evidence of relapse [20].

**Fig. 2:**
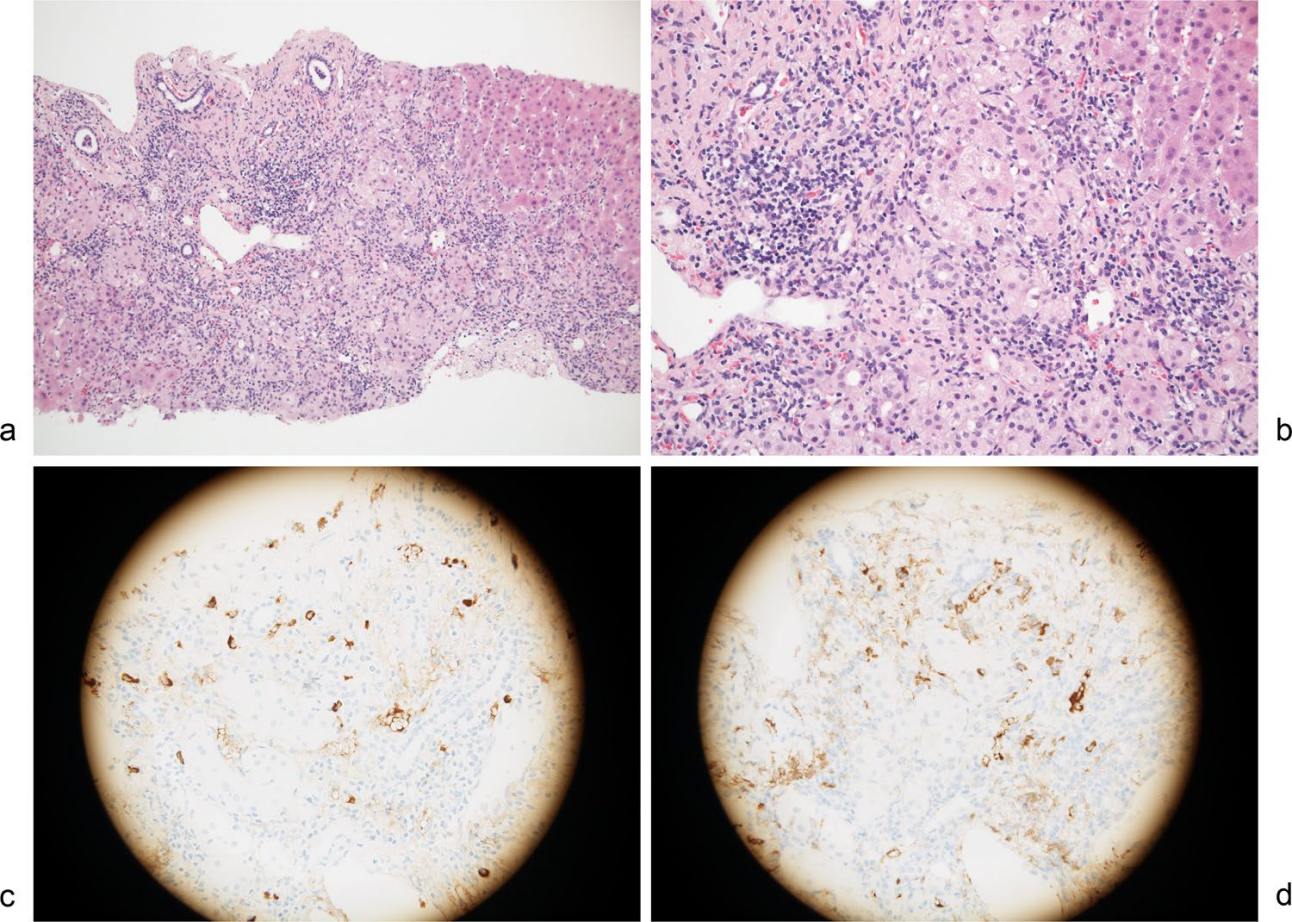
Histopathology of the patient (AIH-73) diagnosed as IgG4-related autoimmune hepatitis (AIH). **a**, **b**: Hematoxylin and eosin staining. Panlobular hepatitis with marked lymphoplasmacytic infiltration that is consistent with AIH. **c**, **d**: Immunostaining for IgG4 (**c**) and IgG (**d**). The numbers of IgG4- and IgG-positive cells were 26 and 29, respectively, in this field

Another case, AIH-73, is a male case in his 50 s, with serum IgG4 of 408 mg/dL. He had IgG4-related sialadenitis and a suspicion of IgG4-SC. Liver biopsy revealed acute hepatitis characteristic of AIH, with significant infiltration of lymphocytes and plasma cells within the portal area (Fig. 2a, b). The numbers of IgG4- and IgG-positive cells were 26 and 29, respectively, in this field (Fig. 2c, d). The findings fulfilled the provisional diagnostic criteria for IgG4-AIH. Notably, there was no evidence of storiform fibrosis or obliterative phlebitis. PSL was highly effective as well and there was no relapse.

### IgG4-hepatopathy

A histopathological analysis of IgG4-hepatopathy was conducted in 20 cases where immunohistochemical staining of liver tissue from patients with IgG4-SC was feasible. The findings revealed that six cases (SC-03, 09, 27, 50, 51, and 60) met the criteria for IgG4-hepatopathy, characterized by significant lymphocyte and plasma cell infiltration in the portal area, the presence of more than 10 IgG4-positive cells/HPF, and more than 40% of IgG4 + /IgG4 ratio. In SC-03, where the number of IgG4-positive cells was notably low (10.6/HPF), many plasma cells were crushed during collection, suggesting the actual count may be higher. In addition, two cases (AIH-56, AIH-60), which were diagnosed as IgG4-AIH by the attending physician but did not meet the diagnostic criteria for IgG4-AIH in this study and were instead classified as potential IgG4-hepatopathy, were included.

**Fig. 3:**
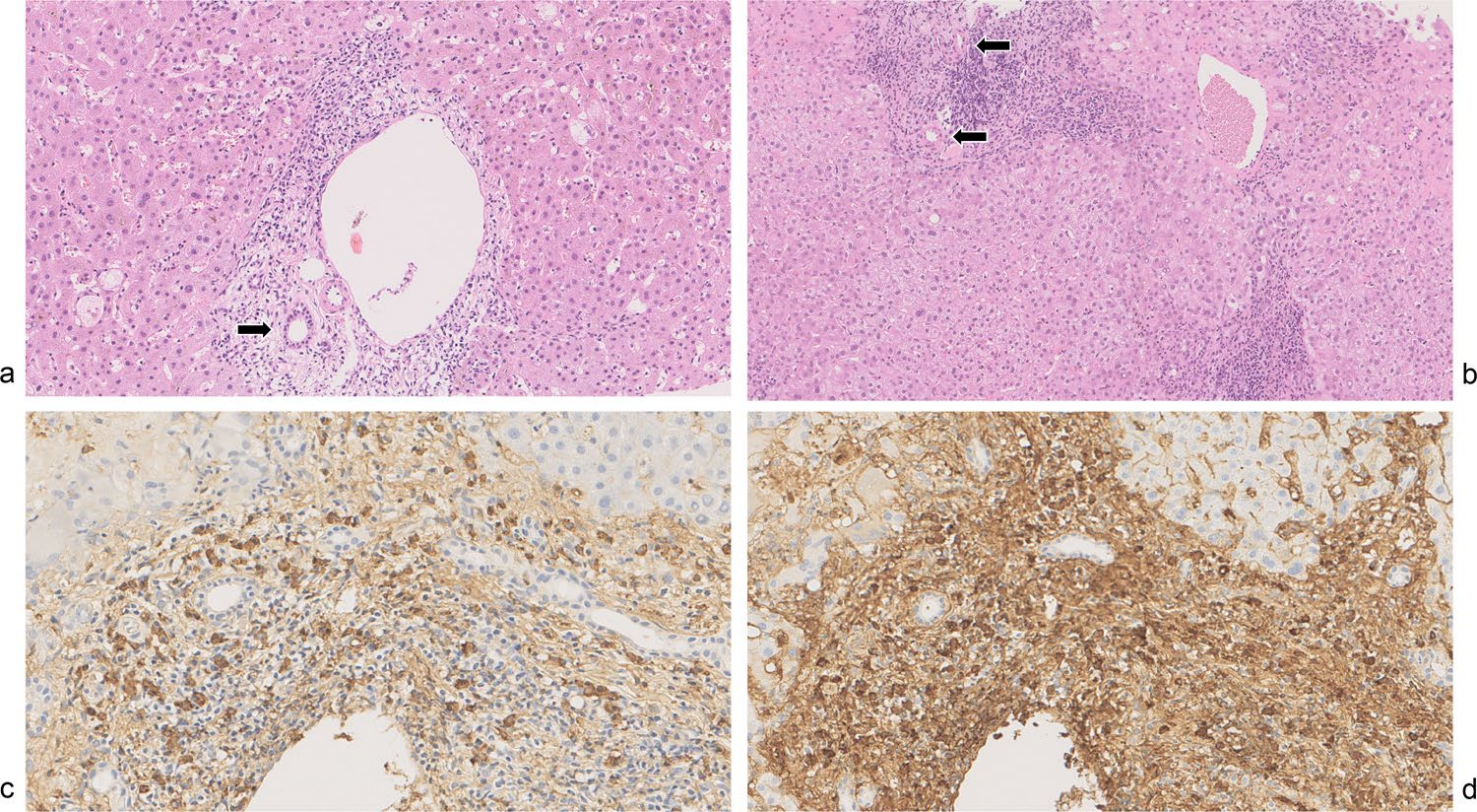
Histopathology of the patient diagnosed as IgG4-related hepatopathy. **a** Hematoxylin and eosin staining. In a typical case (SC-50), marked lymphoplasmacytic infiltration was observed without damaging the interlobular bile ducts (arrow) in the portal area (SC-50). **b** Hematoxylin and eosin staining. In another case (AIH-56) in which the ductular reaction and cholestasis were prominent, the interlobular bile ducts were damaged. Note the interlobular bile ducts adjacent to the interlobular arteries (arrows) were absent. **c**, **d**: Immunostaining for IgG4 (**c**) and IgG (**d**) in SC-50. The numbers of IgG4- and IgG-positive cells were 120 and 155, respectively, in this field, which was isomeric with a high-power field

The results of the histopathological analysis of these three cases are presented in Table 2. Histologically, in addition to lymphocyte and plasma cell infiltration in the portal area, eosinophil infiltration was easily observed in three cases. None of the cases showed evidence of storiform fibrosis or obliterative phlebitis. While some cases exhibited bile duct reactions and cholestasis, the existing intralobular bile ducts were generally preserved, with bile duct abnormalities observed in only one case. Figure 3 presents the liver histopathological findings (HE staining, IgG, and IgG4 staining) from two representative cases (SC-50 and AIH-56) out of the eight analyzed. The portal areas are expanded and exhibit infiltration by lymphocytes and plasma cells, with numerous IgG4-positive cells. A high IgG4 + /IgG + cell ratio is also observed, while the existing interlobular bile ducts remain intact (Fig. 3a; Nakazawa classification Type 2a [21]).

**Table 2:** Histopathological assessment of potential IgG4-hepatopathy cases

Case #	IgG4 + /HPF	IgG4 + /IgG + ratio (%)	Storiform fibrosis	Obliterative phlebitis
SC-03	10.6*	51.6	–	–
SC-09	33.8	54.6	–	–
SC-27	18.5	108.7	–	–
SC-50	71.0	68.9	–	–
SC-51	82.0	223.6	–	–
SC-60	26.0	41.5	–	–
AIH-56	47.3	116.4	–	–
AIH-60	22.0	58.9	–	–

*AIH* autoimmune hepatitis, *HPF OOI* other organ involvement, *IgG4-RD* IgG4-related disease, *NA* not assessable, *AIP* autoimmune pancreatitis, *SC* IgG4-related sclerosing cholangitis

^*^Because the number was counted as 9 due to plasma cell crushing, the real number of plasma cells is very likely to be > 9

Clinical features of these eight patients are shown in Table 3. Seven out of eight cases were male, and all were over 60 years old. Since all cases were extracted from the nationwide survey of IgG4-SC, all eight cases were complicated by IgG4-SC, with two cases also having concurrent AIP. Among the eight cases with IgG4-SC, two were classified as Type 2, two as Type 3, three as Type 1, and one as Type 4, according to the Nakazawa classification [21]. All cases were treated with immunosuppressive drugs, resulting in favorable therapeutic responses.

**Table 3:** Clinical features of potential IgG4-hepatopathy cases

Case #	Gender	At diagnosis							OOI of IgG4-RD	Nakazawa classification^2^	Treatment	Response to treatment
		Age	Bilirubin	ALP (xULN)	AST (U/L)	ALT (U/L)	IgG4	Symptoms				
SC-03	M	63	0.6	2.76	130	137	530	Pruritus	SC	3	PSL	Good
SC-09	M	63	6.9	3.16	111	212	887	Pruritus, icterus	SC	4	PSL	Good
SC-27	M	73	3.0	5.83	448	629	1050	No	SC	1	PSL	Good
SC-50	M	60	4.7	2.17	324	147	–	Abdominal pain	SC	2a	PSL + AZA	Good
SC-51	M	78	9.3	1.52	756	953	370 ^*1^	Icterus	SC	3	PSL	Good
SC-60	M	72	1.8	5.62	228	307	910 ^*2^	No	SC	2b	PSL	Good
AIH-56	M	72	6.6	1.94	85	113	819	Icterus	AIP + SC	1	PSL	Good
AIH-60	F	71	1.0	0.84	137	139	558	No	AIP + SC	1	PSL	Good

Serum IgG4 was shown at 1 year (^*1^) and at 2 weeks (^*2^) of PSL treatment

*AIH* autoimmune hepatitis, *ALP* alkaline phosphatase, *ULN* upper limit of normal, *FU* follow-up, *PSL* prednisolone, *SC* IgG4-related sclerosing cholangitis, *AZA* azathioprine

## Discussion

It is highly improbable that IgG4-AIH would satisfy the "clinical and imaging diagnosis" criteria, which include "diffuse or localized enlargement, mass, nodule, or thickened lesion in one or multiple organs," as outlined in the revised comprehensive diagnostic criteria for IgG4-RD [2]. Consequently, this requirement was excluded from the tentative diagnostic criteria for IgG4-AIH in this study, and instead, the condition of meeting the simplified criteria for AIH [18] was introduced. Elevated IgG4 levels in sera and the pathological criteria were retained. As a result, only two cases met these tentative criteria, suggesting that IgG4-AIH is exceedingly rare when attempting to identify it from among IgG4-SC cohorts, as was done in this study. Nevertheless the possibility remains that the two cases identified in this study may not represent true IgG4-AIH as part of the IgG4-RD spectrum, but rather may be IgG4-RD mimickers. One patient had IgG4-RD affecting other organs, raising the potential for an incidental IgG4-related lesion in the liver. Further follow-up is necessary for this case.

Another potential approach for better understanding the frequency and presentation of IgG4-AIH would be to investigate a cohort of AIH cases that meet the AIH diagnostic criteria, rather than focusing on a cohort of IgG4-SC cases. In cases of AIH, liver biopsy is performed at the time of diagnosis in the majority of cases, potentially increasing the number of eligible cases for investigation. This remains an area for future research and investigation. Currently, the Intractable Hepato-Biliary Diseases Study Group funded by the Ministry of Health, Labour and Welfare is conducting a nationwide study on AIH in Japan. The results of this nationwide survey are expected to facilitate studies of this nature once finalized. On the other hand, when identifying IgG4-AIH from AIH cases, some propose that slightly relaxed pathological criteria should be applied in comparison to the revised comprehensive diagnostic criteria for IgG4-IgG4-RD. In fact, there have been reports suggesting such modifications, including lowering the threshold for IgG4-positive cells to > 5/HPF or an IgG4 + /IgG + cell ratio of ≥ 5% [22–24]. However, in this context, the clinical significance of differentiating IgG4-AIH from classical AIH and diagnosing it as an independent entity remains debatable. For conditions such as AIP and IgG4-SC, there is clear clinical importance in distinguishing them from malignancies like pancreatic cancer and cholangiocarcinoma, respectively. At present, however, there is no strong evidence supporting a similar level of clinical significance in differentiating classical AIH from IgG4-AIH. The only potential relevance may lie in the response to or dependence on corticosteroid therapy. If, for example, IgG4-AIH demonstrates a distinct response to steroids, with sustained remission after treatment discontinuation, it could justify the differentiation from classical AIH, even with relaxed pathological criteria. This remains another important area for further investigation.

In terms of IgG4-hepatopathy, we identified eight cases of IgG4-hepatopathy in the present study, allowing this relatively uncommon condition to be defined as pathological conditions affecting the liver associated with IgG4-RD, especially AIP and IgG4-SC. Umemura et al. described IgG4-hepatopathy characterized by: (1) portal inflammation with or without interface hepatitis, (2) large bile duct obstructive changes, (3) portal sclerosis, (4) lobular hepatitis, and (5) canalicular cholestasis [12]. While we observed similar findings in liver biopsies from patients with IgG4-SC, we specifically focused on the infiltration of a substantial number of IgG4-positive cells as indicative of IgG4-RD. In this study, we defined IgG4-hepatopathy based on a three-field average showing more than 10 IgG4-positive cells/HPF and an IgG4/IgG-positive cell ratio exceeding 40%, indicating significant lymphocyte infiltration in the portal area. Meanwhile, Naitoh et al. reported that IgG4-positive cell infiltration in the portal region was present in 5 out of 19 patients with small bile duct involvement related to IgG4-SC [25]. According to their study, four out of five cases with small bile duct involvement were classified as Type 2 of Nakazawa classification, while one case was classified as Type 3, suggesting that small bile duct involvement is commonly observed in IgG4-SC cases with intrahepatic bile duct involvement. A similar trend was noted in the IgG4-hepatopathy cases studied here. Based on these similarities, the small bile duct involvement described by Naitoh et al. and IgG4-hepatopathy may represent analogous pathological conditions. However, while bile duct damage reported by Naitoh et al. was observed in only one case in our cohort, IgG4-SC is typically characterized by the absence of bile duct epithelial damage, being consistent with the characteristics of IgG4-RD. It may be necessary to further verify the potential for large bile duct stenosis caused by IgG4-SC to secondarily affect the interlobular bile ducts in a larger cohort of cases.

IgG4-hepatopathy may also play a crucial role in the diagnosis of IgG4-SC. When IgG4-SC is suspected based on serological and imaging studies, obtaining histological evidence through biliary biopsy can be challenging. However, if a liver biopsy is conducted and reveals more than 10 IgG4-positive cells/HPF along with indications of IgG4-hepatopathy, these findings may substantiate the diagnosis of IgG4-SC. Thus, liver biopsy may have significant clinical implications in cases where IgG4-SC is suspected. Conversely, several unresolved questions remain concerning IgG4-hepatopathy, including whether liver damage resolves or persists following the treatment of AIP or IgG4-SC. In addition, it is unclear whether IgG4-related diseases outside the biliopancreatic region, such as those affecting the lacrimal and salivary glands, also manifest in similar ways.

In summary, this study identified 2 cases of IgG4-AIH and 8 cases of IgG4-hepatopathy through a clinicopathological examination of a cohort comprising 1,096 cases of IgG4- SC. As previously noted, a clinicopathological investigation of IgG4-AIH should also be conducted within the AIH cohort, as it would be particularly insightful to determine whether such an investigation can uncover clinically meaningful differences between IgG4-AIH and classical AIH. Moreover, the findings of this study suggest that the presence of IgG4-hepatopathy may hold significance in the diagnosis of suspected IgG4-SC.

## Abbreviations

IgG4-RD: IgG4-related disease
HPF: High-power field
AIH: Autoimmune hepatitis
IAIHG: International AIH Group
AIP: Autoimmune pancreatitis
IgG4-SC: IgG4-related sclerosing cholangitis
IgG4-AIH: IgG4-related autoimmune hepatitis
